# Health Contributing Factors in Higher Education Students: The Importance of Family and Friends

**DOI:** 10.3390/healthcare6040147

**Published:** 2018-12-14

**Authors:** Santiago Yubero, Raúl Navarro, Elisa Larrañaga, Macarena Esteban, Jesús Gutiérrez, María Elche

**Affiliations:** 1 Department of Psychology, University of Castilla-La Mancha, 16071 Cuenca, Spain; santiago.yubero@uclm.es (S.Y.); Jesus.Gutierrez@uclm.es (J.G.); maria.elche@uclm.es (M.E.); 2 Department of Education and Social Psychology, Pablo de Olavide University, 41013 Sevilla, Spain; mestiba@upo.es

**Keywords:** university, well-being, friends, family, health, health promotion

## Abstract

The aim of this study was to determine well-being and its relationship to social support from friends and family communication in university students. A cross-sectional study was conducted with 1679 university students aged 18–25 years from four universities in Spain. Logistical regression models were tested (*p* < 0.05). The students in the last year of university reported higher well-being scores in comparison with students in the previous years. Well-being was inversely related to family communication problems. Family communication and social support from friends were the factors that associated the most with better well-being. All the regression models were statistically significant and explained from 16% of the well-being in Year 4 students until 34% of the well-being in Year 1 students. Our findings could be useful for developing interventions to promote health in order to improve college students’ well-being. It is important for strategies to be developed in mental and family health areas.

## 1. Introduction

According to the World Health Organization, health is a state of complete physical, mental, and social well-being, and not merely the absence of disease or illness. This definition has resulted in an increasing number of research studies that have analyzed well-being. However, compared to the data available on adolescents [1], few studies have been conducted about university students’ subjective well-being. This differentiated population [2] is under psycho-social pressure that obliges university students to respond to high academic and social demands [3]. Indeed it has been found that university students are very vulnerable to stress periods when they are freshmen [4,5].

Health promotion has been related to the family, which acts as an important group in adolescence. For example, family relationships have proven relevant for social, cognitive, and emotional adjustment [6,7,8]. Family relationships are among the factors that are more closely related to quality of life in adolescence [9], but also to engage in risk behaviors when such relationships are problematic. These associations have also been found among university students [10,11,12]. Consequently, the quality of family relationships and communication impacts adolescents’ mental health [13]. In their systematic review, Serafini et al. [14] indicate that most studies report a strong association between adversities and suicidality, including adverse family life events, such as social problems of family members, a poor family environment, and interpersonal difficulties with peers [15,16,17,18]. In contrast, McKeown et al. [19] found that family cohesion was a significant protective factor.

Nowadays, university students live a prolongation of their emerging adulthood, which involves increased dependence on the family [20] and normally involves satisfactory relationships with their parents [21]. Friedlander et al. [4] have shown that, even when university students are under stress, they better integrate into university when they feel supported by their families. Research has also shown that support from friends also contributes to improve well-being and psycho-social adjustment [22,23]. For example, research has documented that social support from friends has an inverse impact on the anxiety that university students experience [24].

Inquiry to determine whether family communication and friends’ support are related to university students’ subjective well-being is still needed to include these elements in policies and strategies that aim to promote health among higher education students. Therefore, the objective of this study was to analyze the relationship linking subjective well-being, family communication, and social support from friends in a sample of university students. Good communication between family members and receiving social support from friends was expected to be positively associated with the subjective well-being of young university students in Spain.

## 2. Methods

### 2.1. Participants

The sample was comprised of 1679 university students from Spain. Their ages ranged from 18 to 25 years (M = 20.71; SD = 1.88). Of these, 32.3% were Year 1 students, 29.7% were Year 2 students, 24.1% were Year 3 students, and 14% were Year 4 students. In gender terms, 14.5% were male and 85.5% were female.

### 2.2. Instruments

#### 2.2.1. Subjective Well-Being

The Spanish version of the Satisfaction with Life Scale (SWLS) [25], adapted by Atienza et al. [26], was used to evaluate subjective well-being. The scale was comprised of five items scored on a five-point scale (0 = *very dissatisfied* to 5 = *very satisfied*). The results obtained in Spanish samples support a single-factor structure [27]. In the present study, Cronbach’s alpha reliability was 0.85.

#### 2.2.2. Social Support from Friends

The employed scale was the Spanish version [28] of the Perceived Acceptance Scale (PAS) [29] by selecting the items related to friend relationships. The participants used a Likert-type scale ranging from 1 (*never*) to 5 (*always*) to answer how often each item describes what (s)he thinks and/or how (s)he feels. The scale’s Cronbach’s alpha reliability coefficient was 0.89.

#### 2.2.3. Family Communication

The Spanish version by Musitu et al. [30,31] of the parent–child communication scale [32] was used to assess university students’ perceptions of communication with their parents. The scale contained 20 items, which scored on a five-point Likert-type scale (1 = never to 5 = always). The scale comprises three dimensions in the Spanish version that measure open communication (e.g., “My parent tries to understand my point of view”), offensive communication (e.g., “My parent insults me when they are angry with me”), and avoidant communication (e.g., “There are topics that I avoid discussing with my parents). Open communication had a high level of reliability, α = 0.86; the reliability level was lower for avoidant communication, α = 0.55, and for offensive communication, α = 0.65.

### 2.3. Design

The present study is a cross-sectional survey-based study.

### 2.4. Procedure

The research project was approved by the Spanish Ministry of Economy and Competitiveness (no. 70822). The data collection procedure met the requirements set out in the Code of Good Scientific Practices approved by the Spanish Higher Council for Scientific Research (CSIC, its acronym in Spanish) in March 2010. Participants were students from public universities from randomly selected four Spanish autonomous communities (class was the sample unit). Data were collected during academic year 2016/2017. Questionnaires were anonymous and filled out in class during class hours at the end of teaching sessions. Students were provided with information on the study and given the same instructions. Data confidentiality was also guaranteed.

### 2.5. Data Analysis

Firstly, an inferential analysis of the study variables was performed by gender and academic year. Secondly, correlational analyses among the study variables were conducted. Thirdly, a logistic regression analysis was performed by academic year, and included gender as a control variable. All the analyses were conducted using 95% confidence intervals (95%CI). Version 23 of the SPSS software was used in the data analysis.

## 3. Results

Regarding well-being, 5.3% of males and 7% of females obtained a score below the mean (2.5), without significant differences (χ^2^ = 0.95; *p* = 0.203). No significant differences were found in gender distribution terms according to each academic year (χ^2^ = 4.69; *p* = 0.196). As far as academic year was concerned, the proportion of students with low levels of subjective well-being was slightly higher among those students of Years 1 and 4, but contingence was not significant (Year 1: 8.1%; Year 2: 6.8%; Year 3: 4.0%; Year 4: 8.1%, χ^2^ = 7.32; *p* = 0.062).

The inferential analysis (Table 1) showed that females felt more supported by friends (*p* < 0.05) and males reported more avoidant communication with their parents (*p* < 0.01). The average level of well-being among university students was higher among the students of Years 3 and 4 (*p* < 0.01). Regarding family communication, the students of Year 1 showed more avoidant communication (*p* < 0.01).

The correlational analyses (Table 2) showed that open communication with parents and social support from friends were positively related with subjective well-being. Conversely, negative family relationships, measured by offensive and avoidant communication with parents, were negatively related with subjective well-being.

The results of the logistic regression analyses are reported in Table 3. An association was found to link friends’ support (OR = 2.99; 95%CI = 2.26–3.97), open family communication (OR = 2.05; 95%CI = 1.49–2.84), and the subjective well-being of our university students. When analyzing the variables by academic year, the significance of both variables was maintained for Year 1; friends’ support (OR = 4.21; 95%CI = 2.62–6.78) and open family communication (OR = 2.24; 95%CI = 1.29–3.89 explained the variance in subjective well-being, which came to 34%. The same association was found to link subjective well-being, friends’ support (OR = 3.99; 95%CI = 1.78–8.92), and open family communication (OR = 3.49; 95%CI = 1.41–8.60) among the Year 3 students, which explained 31% of variance. In Year 2, subjective well-being was related only with open family communication (OR = 1.85; 95%CI = 1.03–3.33) and the statistical significance of family communication disappeared in Year 4, with subjective well-being being associated only with friends’ support (OR = 3.50; 95%CI = 1.70–7.20).

## 4. Discussion

University involves a process of vital change. This study examined the association between subjective well-being among university students and their relation with social support from friends and family communication. Subjective well-being in adolescence has been related with family relationships [9] and friends’ support [22,23]. This study has focused on analyzing if both variables were also related with subjective well-being in subsequent years, when individuals attend higher education studies. Between 5% and 8% of university students reported a low level of subjective well-being, with no significant differences found for gender or academic year. This result confirms that university students are vulnerable [3,4,5], and it shows the importance of paying attention to the mental health of higher education students beyond Year 1, as previous research has shown [4,5]. 

The results showed that university students’ subjective well-being was inversely related to family communication problems, and was directly related to social support from friends and open family communication. In line with previous studies, the present results show that positive family relationships [21] and open family communication prevailed over communication problems as well as avoidant and offensive communication among university students. However, the females in the present study reported a more positive environment, with more friends’ support and less avoidant communication with their parents. These results coincide with former research conducted with adolescents, in that females maintain better relationships with their peers [33] and their parents [34]. According to year, the students of Year 1 reported poorer subjective well-being. These results match those obtained in previous research, which report how hard it is to fit in the university context [4,5].

From a statistical point of view, gender did not come over as being significant in the regression models, nor were family communication problems significant. Open family communication was significant for the university students of Years 1, 2, and 3. However, open family communication was not significant in the model of the Year 4 students. This result could be seen as a milestone in students’ independence of their families. In their last university year, they are getting ready to plunge into the world of work, which necessarily means the end of family dependence. The results obtained from the Year 1 students match those from previous research works, which have shown the importance of family to duly fit into university [4] and its link to mental health [13]. The results obtained from the students of the other academic years showed the importance of having positive family relationships throughout university life. Although many university students have to move to another city to attend their university courses, they maintain links with their families and friends. Nevertheless, some parents see no need to keep paying attention to their offspring when they start their university lives [35] and are unaware of the positive effect of communication with them when they go to university. Although certain previous studies have already shown that it is important for the family to make fitting into university easier for freshmen [36], our results revealed that the family is important not only in the first university years. Additionally, parents may also benefit from information on the relevance of the role they play in their children’s well-being by maintaining open communication with them.

Social support from friends was significant among the university students of Years 1, 3, and 4. Other studies have already shown that, in certain specific contexts, well-being can be associated more with the social support perceived by individuals [37]. The obtained results show the importance of feeling supported by friends at university to feel well-being. Previous studies have already demonstrated the importance of friends’ support to protect against the anxiety experienced by university students, and to contribute to well-being in adolescence [22,23]. Nevertheless, it is difficult to explain the lack of significance of friends’ support for the Year 2 students. This study needs to be replicated with larger samples. It would be advisable to conduct certain qualitative studies to allow us to identify the reasons for this result.

This study has limitations that should be taken into account. Sample composition is one of them. It will be necessary to increase the number of male participants to guarantee the representativeness of the results by gender. Secondly, measuring the variables via self-report is another limitation, as it may lead to a certain level of desirability. Thirdly, the analyses in this study are cross-sectional and correlational, so no inferences about causality or directionality can be made. Fourthly, it would be necessary to conduct qualitative studies to understand how the importance of the social context evolves in relation to university students’ well-being throughout their academic lives. In subsequent studies, it will be interesting to compare the family relationships of those students who live with their parents and those who live away from their family context, for example, in a hall of residence or with other students. Today, Web 2.0 promotes social interactions and makes them easier [38], regardless of individuals’ locations and the distance between them. It may also be interesting to analyze the way interactions are established through the Internet and how these relationships are related with subjective well-being.

The Strategy of Health Promotion and Prevention of the Spanish National Health System includes actions in the educational environment and in the field of well-being and emotional health. The university context is also an educational environment that requires actions to promote and prevent students’ well-being and mental health. This study evidences the associations that link family relationships, social support from friends, and the well-being of university students throughout their academic lives. It can be concluded that a good network of social support is necessary to guarantee the ability to deal with the changes that university students face throughout their university years. The key role played by families and friends as a main support should also be highlighted. It is essential to bear these results in mind to increase university students’ well-being by focusing on promoting health and prioritizing the relationship between youths and their family and social environment.

We conclude that having a good social support network available is necessary to ensure that university students have the capacity to face the various changes they must confront when they go to university and, above all, the fundamental role that family and friends play as their main sources of support. Family communication acts as an indicator of the quality of a family relationship, and is the main resource that parents can rely on to favor their offspring’s social adjustment [39], not only in infancy and adolescence but also in university life. The results confirm good family communication during university life correlates with positive peer relationships, which has already been reported in adolescents [40]. Consequently, the optimum family climate for youths to develop in is that in which affection and open communication prevail and in which discussion to exchange points of view and to adopt one’s own views is allowed. It is important for the family to talk about matters that worry youths, share personal feelings and concerns with sons and daughters, and show a receptive attitude. This involves making interactions with family and friends with fondness and acceptance that are based on the capacity to put oneself in another person’s place in order to suitably respond to an interaction by creating affect, personal security, trust, integration, stability, and cohesion.

## Figures and Tables

**Table 1:** Differences in subjective well-being, social support from friends, and family communication per gender and academic year.

Variables	Total	Gender			Academic Year				
		Males (*n* = 244)	Females (*n* = 1435)	t	1 (*n* = 542)	2 (*n* = 497)	3 (*n* = 405)	4 (*n* = 235)	F
Subjective well-being	3.68	3.71	3.67	0.67	3.60	3.67	3.79	3.70	5.07 **
Friends Support	4.10	4.01	4.12	−2.24 *	4.06	4.14	4.13	4.10	1.41
Open communication	3.90	3.91	3.90	0.15	3.86	3.91	3.95	3.92	1.32
Offensive communication	2.06	2.01	2.07	−1.10	2.09	2.08	2.05	1.96	1.99
Avoidant communication	2.69	2.83	2.67	2.99 **	2.78	2.71	2.61	2.60	5.07 **

* *p* < 0.05, ** *p* < 0.01.

**Table 2:** Correlational analyses among the study variables.

Study Variables	1	2	3	4	5
1. Subjective well-being	--	0.374 ***	0.385 ***	−0.230 ***	−0.189 ***
2. Friends support		--	0.251 ***	−0.138 ***	−0.131 ***
3. Open communication			--	−0.484 ***	−0.447 ***
4. Offensive communication				--	0.386 ***
5. Avoidant communication					--

*** *p* < 0.001.

**Table 3:** Logistic regression analyses examining the associations linking subjective well-being, social support from friends, and family communication per academic year, with gender as a control variable.

Study Variables	Total		Year 1		Year 2		Year 3		Year 4	
	OR	95%CI	OR	95%CI	OR	95%CI	OR	95%CI	OR	95%CI
**Gender**	0.58	0.29–1.17	0.66	0.21–2.10	0.88	0.28–2.71	0.00	0.00–0.00	0.97	0.19–4.93
**Social support from friends**	2.99 ***	2.26–3.97	4.21 ***	2.62–6.78	1.60	0.96–2.67	3.99 ***	1.78–8.92	3.50 ***	1.70–7.20
**Open Communication**	2.05 ***	1.49–2.84	2.24 **	1.29–3.89	1.85 *	1.03–3.33	3.49 **	1.41–8.60	1.28	0.52–3.14
**Offensive communication**	0.96	0.70–1.32	0.83	0.45–1.53	1.02	0.60–1.71	1.09	0.46–2.62	1.01	0.43–2.37
**Avoidant communication**	0.78	0.56–1.07	0.84	0.48–1.47	0.66	0.37–1.19	0.76	0.33–1.77	0.72	0.31–1.69
**χ^2^**	141.49 ***		80.25 ***		23.48 ***		35.25 ***		15.99 **	
**R^2^ Nagelkerke**	0.22		0.34		0.18		0.31		0.16	

95%CI: 95% confidence interval; OR: odds ratio; * *p* < 0.05, ** *p* < 0.01, *** *p* < 0.001.
